# Understanding plasticity of chemotaxis in *C. elegans*, a computational model of associative learning

**DOI:** 10.1186/1471-2202-13-S1-P162

**Published:** 2012-07-16

**Authors:** Tom Sanders, Netta Cohen

**Affiliations:** 1 School of Computing, University of Leeds, Leeds, West Yorkshire, LS2 9JT, UK

## 

The nematode *C. elegans* has been a model organism for over 40 years. It is the only multicellular organism for which we know the full genome and the complete cell lineage from zygote to adult. In addition, the connectivity diagram of its nervous system has been worked out to the level of individual synapses. Together, this makes *C. elegans* ideal for a wide variety of studies focussing on its genetics, behaviour or its nervous system.

*C. elegans* uses chemical cues to steer its orientation towards food sources and away from threats. This form of behaviour, chemotaxis, is done using two distinct strategies: (1) biased random walk or so called pirouette strategy and (2) slight steering or the weathervane strategy. While many of the sensory neurons have been characterised in terms of the specific chemicals they detect, the sites of sensory integration downstream of these neurons and the neuronal pathways of these two strategies have yet to be discovered. Furthermore, chemotaxis is subject to plasticity: The strength of the chemotactic response to a chemical and even its polarity can be plastic and amenable to habituation, sensitization and multiple forms of short and long term memory. These learned responses can be unlearned and are selective to one chemical.

We present a computational model of chemotaxis to NaCl and related associative learning in *C. elegans.* Our model of chemotaxis (before learning) is an extension of a previously published model. To ensure realistic behaviour and computation, neuronal parameters are set using behavioural and calcium imaging data, and detailed analysis of the model. Using multiple virtual environments, cellular or synaptic plasticity and a single or two pathways controlling the chemotaxis strategies, we achieve an understanding of the limits of each site of plasticity under different constraints. By comparing our data with behavioural data from associative learning studies in the biological worm, we show that synaptic plasticity fails to explain all results, suggesting a role for cellular plasticity. Furthermore, our model shows differential effects depending on whether a single circuit pathway or two distinct pathways control the two chemotaxis strategies, leading to a straight forward behavioural prediction.

Further research will focus on adding sensory neurons to investigate the integration of different sensory signals. Apart from further explaining chemotaxis, genetic studies have shown that some forms of learning depend on multiple sensory pathways, making this a logical next step. The end goal is to create a complete model of the nervous system of *C. elegans* to serve in the understanding of its ‘brain’ and behaviour from a systems perspective. Our study shows the feasibility and importance of an *in silico* methodology towards understanding sensorimotor pathways and sensory plasticity in *C. elegans* and in higher animals.

